# Nasolabial cyst: diagnostic and therapeutical aspects

**DOI:** 10.1016/S1808-8694(15)30749-7

**Published:** 2015-10-19

**Authors:** Romualdo Suzano Louzeiro Tiago, Mayko Soares Maia, Gustavo Motta Simplício do Nascimento, Juliano Piotto Correa, Daniel Cauduro Salgado

**Affiliations:** 1 Doctor in Sciences, graduate program on Otorhinolaryngology and Head & Neck Surgery, Sao Paulo Federal University. Assistant physician, Otorhinolaryngology Unit, HSPM; 2 Medical resident, Otorhinolaryngology Unit, HSPM; 3 Medical resident, Otorhinolaryngology Unit, HSPM; 4 Medical resident, Otorhinolaryngology Unit, HSPM; 5 Medical resident, Otorhinolaryngology Unit, HSPM. Otorhinolaryngology Unit, Hospital for City Workers, Sao Paulo – HSPM

**Keywords:** cyst, diagnosis, enucleation, nose

## Abstract

Nasolabial cyst is a rare lesion situated behind the ala nasi, extending backwards into the inferior nasal meatus and forward into the labio-gingival sulcus.

**Aim:**

We present our case of a nasolabial cyst, with the purpose of discussing clinical presentation, diagnosis and the more suitable surgical techniques to treat this disorder.

**Materials and methods:**

A retrospective study of eight patients with diagnosis of nasolabial cyst, carried out in the period of january/2000 to december/2006. The diagnosis was suggested by otorhinolaryngology exam and computer tomography. All patients were submitted to surgical treatment (enucleation) and definitive diagnosis was confirmed by histopathology.

**Results:**

Predominant symptoms were nasal obstruction, swelling in the nasal vestibule region and local pain. Patients had had symptoms for a median of 26.2 months. CT scan was performed in all patients, showing a well outlined cystic lesion with bone remodeling in some cases. Median sizes of the cysts were 2.18cm. There was no evidence of recurrence during a mean follow-up of 19.5 months.

**Conclusion:**

Nasolabial cysts are rare lesions. Common presentation is a well-confined swelling, local pain and nasal obstruction. Enucleation is the treatment of choice with low recurrence rate.

## INTRODUCTION

Nasolabial cysts are uncommon lesions located close to the alar cartilage of the nose, extending into the lower nasal meatus, the upper gingivolabial sulcus and the floor of the nasal vestibule.^1^^,^^2^

Zuckerkandl^1^^,^^3^ described nasolabial cysts in 1882. There are many synonyms: nasoalveolar cyst, nasal vestibular cyst, nasal wing cyst and Klestadt's cyst.^2^^,^^4^^,^^5^ Rao^4^ revised the nomenclature and defined nasolabial cysts as lesions located entirely within soft tissue, different from nasoalveolar cysts, which cause maxillary bone erosion.

The pathogenesis of nasolabial cysts is not fully understood. Two hypotheses are currently accepted: they originate from facial fissure cysts or from remnants of the nasolacrimal ducts. The former hypothesis suggests that these cysts derive from sequestering of embryological epithelial tissue in facial fissures resulting from fusion of the maxillary and nasal processes (lateral and medial).3 The latter hypothesis suggests that persisting nasolacrimal duct epithelial remnants located between the maxillary and nasal processes gives rise to nasolabial cysts.^4^

Nasolabial cysts are found most often in female adults in the fourth to fifth decades of life. They commonly present as a localized painless swelling in the nasogenian sulcus and the nasal alar base.^2^ Diagnostic tests include flexible nasofibroscopy, computed tomography (CT) and magnetic resonance imaging (MRI). Treatment is surgical, usually cyst marsupialization or enucleation.^2^^,^^4^, ^5^, ^6^, ^7^, ^8^, ^9^, ^10^ The recurrence rate varies according to the technique, but it is generally low.

The aim of this paper was to assess a nasolabial cyst series to describe the clinical presentation, the diagnosis and the appropriate surgical techniques used in this disease.

## MATERIAL AND METHOD

A retrospective study was made of eight nasolabial cyst patients diagnosed between January 2000 and December 2006. The institution's Research Ethics Committee approved the research project (number 73/2007). Nasolabial cysts were diagnosed based on the otolaryngological exam and CT imaging. All of the patients underwent cyst enucleation surgery; histopathology confirmed the diagnosis. Collected data included the sex, age, race, clinical findings, duration of the disease, tests, cyst location, cyst size, surgical procedure, histopathology, postperative follow-up, and recurrence.

## RESULTS

There were eight female patients with a mean age of 47.6 years. There were no differences related to the side in which cysts arose, or to race. One of the patients (number 5) presented bilateral nasolabial cysts and another (number 6) had a history of bilateral cysts; in this latter case, only the cyst that recurred after marsupialization was taken into account. There were, therefore, nine nasolabial cysts in our analysis (Table 1).

**Table 1:** Age, sex, side in which the cyst was located, and race data.

Patient	Age	Sex	Side	Race
1	49	F	R	White
2	33	F	L	Black
3	20	F	R	White
4	61	F	L	Black
5	43	F	R/ L	Black
6	67	F	Ra/ Lb	White
7	45	F	R	White
8	63	F	L	Black

**Key:** F = female; R = right; L = left; a = recurrence of previous marsupialization; b = prior history of enucleation

The predominant symptoms were: nasal obstruction, swelling in the nasal vestibule, and pain upon local palpation with no signs of infection. The mean time between the onset of symptoms and a consultation with a specialist was 26.2 months. CT was done in all of the patients, showing well-defined cysts in the deep lateral nasal region. Bone remodeling resulting from compression due to a cyst was seen in some of the cases. The mean diameter of cysts was 2.18 cm. Surgical enucleation under general anesthesia through a sublabial incision was done in all of the cases. Histopathology was done in all of the surgical specimens to confirm the diagnosis. The mean postoperative follow-up was 19.5 months; none of the cases recurred (Table 2).

**Table 2:** Symptoms, duration of disease, CT findings, size of cyst, type of surgery, postoperative follow-up, and recurrence data.

Patient	Symptom	Duration of disease	Computed tomography	Size	Type of surgery	Follow-up	Recurrence
1	Nasal obstruction / pain	1 month	Cyst	1,3 cm	Enucleation	10 months	No
2	Swelling / pain	12 months	Cyst / bone remodeling	2,5 cm	Enucleation	45 months	No
3	Headache	1 month	Cyst	2,5 cm	Enucleation	–	No
4	4 Nasal obstruction / swelling	6 months	Cyst / bone remodeling	3 cm	Enucleation	16 months	No
5	Nasal obstruction / coriza / hyposmia	4 months	Bilateral cyst / bone remodeling	R – 2,5 cm L – 2 cm	Enucleation	14 months	No
6	Nasal obstruction	60 months	Cyst	1,3 cm	Enucleation	57 months	No
7	Nasal obstruction / swelling	120 months	Cyst / bone remodeling	2,5 cm	Enucleation	3 months	No
8	Swelling	12 months	Cyst	2 cm	Enucleation	2 months	No

**Key:** R = right; L = left; – = no information

## DISCUSSION

**Figure 1:**
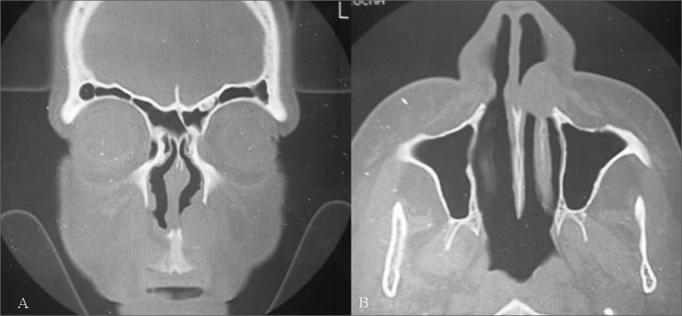
Radiological findings (computed tomography) of a nasolabial cyst – coronal section (A) and axial section (B) of a left unilateral cyst.

Nasolabial cysts are rare, comprising about 0.3% of maxillary cysts.^6^^,^^8^ The study sample included females aged above the third decade of life (mostly fourth and fifth decades). Cysts were unilateral in 85% of cases (Figure 1). A 3.5:1 female to male ratio in the incidence of nasolabial cysts has been noted in the literature; most of these cysts occur between the fourth and fifth decades of life, and are unilateral in 90% of cases.^4^, ^5^, ^6^

The mean age at which cysts were detected in our study was 45.5 years, similar to other published results.^4^^,^^5^^,^^9^^,^^10^ Nasolabial cysts, probably due to their slow growth, tend to be detected in older patients. There was no ethnic predilection in our sample. Schuman^4^ has reported no race preference in nasolabial cysts. There was no difference in cyst location to the right or left, again similar to other published data.^4^^,^^11^

A few nasolabial cyst patients may be asymptomatic, but most present at least one of three main symptoms: partial or total nasal obstruction, localized swelling or local pain.^4^^,^^5^^,^^11^^,^^12^ The main symptoms in this study were: nasal obstruction (62.5%), swelling in the nasal vestibule (50%) and pain upon palpation (25%). Graamans et al.^6^ have reported that a well-located fluctuating swelling with a cystic consistency in the nasolabial sulcus is a definitive sign of a nasolabial cyst. The mean time between the onset of symptoms and a consultation with a specialist was 26.2 months. Schuman^4^ reported that 65% of the patients had symptoms for over 12 months before a diagnosis was made.

The differential diagnosis includes oronasal cysts in general, particularly the nasopalatine cyst, which is the most common maxillary non-odontogenic cystic lesion.^13^ The physical examination demonstrates swelling in the hard palate, and CT shows a well-defined rounded or oval lesion in the mid-maxillary area.^13^

**Figure 2:**
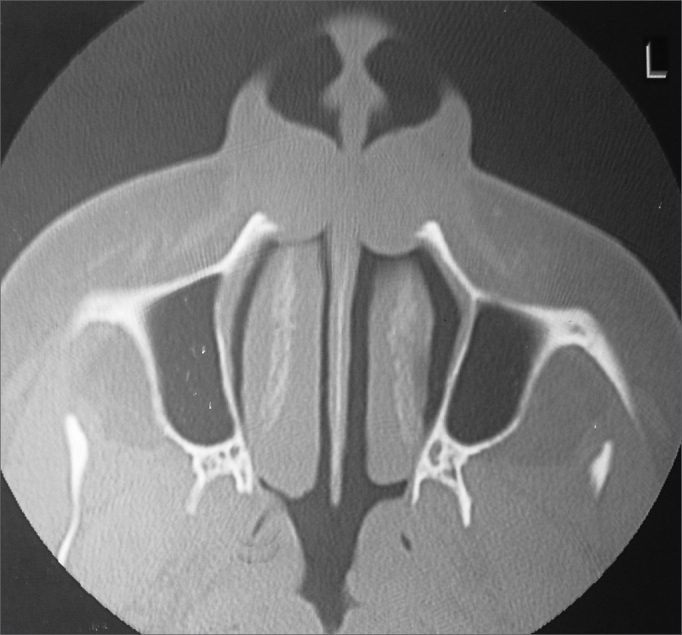
Radiological findings (computed tomography) of a nasolabial cyst – axial section of bilateral cysts.

CT or MRI reveal the soft-tissue origin of nasolabial cysts, which avoids unnecessary dental surgery or needle aspiration.^7^ CT usually shows a homogeneous, non-contrast enhancing cystic lesion^11^^,^^14^ anterior to the pirifom opening; remodeling of the underlying maxillary bone may be seen in larger cysts.^11^ CT was done in all of our sample patients, demonstrating well defined cystic lesions in deep lateral nasal areas; in some cases there was maxillary bone remodeling (Figure 2). The mean diameter of cysts on CT was 2.18 cm, similar to those reported by other authors.^6^^,^^8^^,^^14^ Nasolabial cysts appear on MRI as homogeneous intermediate intensity T1 signals and homogeneous high intensity T2 signals, similar to glandular odontogenic cysts and radicular cysts.^15^ MRI is extremely useful in the differential diagnosis between nasolabial and nasopalatine cysts. The latter presents homogeneous high intensity T1 and T2 signals.^16^ CT is less costly, compared to MRI, and is our preferred option in the diagnosis of nasolabial cysts.

Surgical enucleation is the preferred treatment reported in most of the published papers.^2^^,^^5^, ^6^, ^7^, ^8^ Other methods include: needle aspiration, cauterization, injecting sclerosants, and incision for drainage and marsupialization. These alternative methods, however, have high recurrence rates.^11^

**Figure 3:**
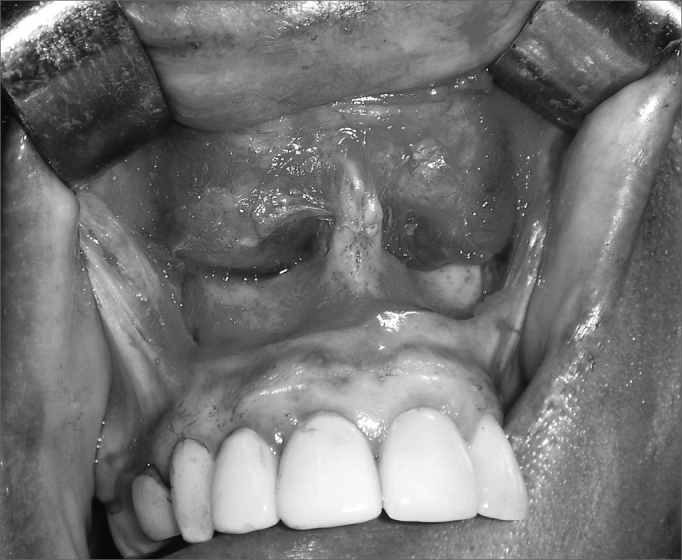
Sublabial approach for resecting a bilateral nasolabial cyst, remodeling of the lower ridge of the piriform opening may be seen.

In this study we used the intra-oral enucleation technique with a sublabial approach followed by dissection along surgical planes to the piriform opening (Figure 3). Cysts were completely removed; in some cases a portion of the floor of the nasal vestibule that had adhered to the capsule of the cyst was resected. In such cases a dressing with topical antibiotics was applied and the floor of the vestibule was allowed to heal by second intention to avoid stenosis due to scarring.

Su et al.^9^ investigated endoscopic marsupialization as a new approach in the treatment of nasolabial cysts. These authors opened the cyst amply along the floor of the nasal vestibule under local anesthesia. The series was composed of 16 patients, of which 15 underwent endoscopic marsupialization; the cyst was not found in one patient, who underwent sublabial enucleation.^9^ No recurrences were seen in the mean 16-month follow-up period.^9^ Su et al.^10^ noted one recurrence in a more recent study of endoscopic marsupialization in a group of 10 patients, monitored for a mean period of 16 months.

**Figure 4:**
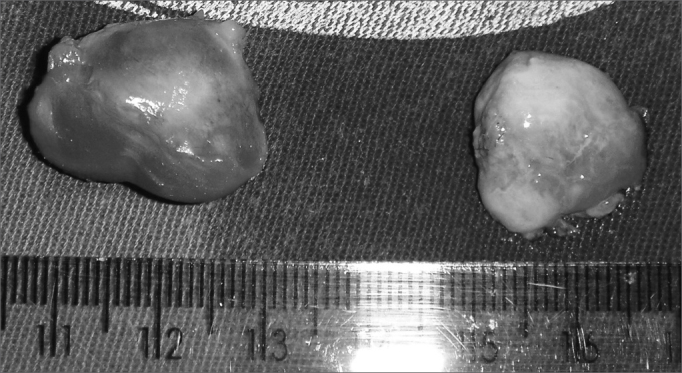
Aspect of a bilateral nasolabial cyst after surgical enucleation.

Histopathology reveals a ciliated pseudostratified columnar epithelium and occasionally a stratified squamous epithelium lining the cystic lumen.^5^ Su et al.^10^ studied the inner surface of these cysts by electron microscopy, which showed a non-ciliated columnar epithelium associated with basal cells and mucous-producing cells (goblet cells). Histopathology was done in all of our surgical specimens (Figure 4); the general description was a cystic lesion with signs of chronic inflammation, a fibrous capsule, a smooth bright inner surface, and a yellowish seromucous liquid content.

The mean postoperative follow-up period was 19.5 months, during which there were no recurrences. Most of the authors have not described a follow-up period, suggesting that total excision of the cyst is curative, and that recurrence is rare.^4^^,^^5^^,^^12^ One of the patients in the present study (number 6) had a history of bilateral cysts that hade been treated by enucleation (to the left) and marsupialization (to the right); in this case, recurrence was on the right ten years after surgery. Su et al.^10^ reported one recurrence in a group of 10 patients treated by endoscopic marsupialization in which the mean follow-up period was 16 months (8–65 months). We believe that longer follow-up periods are needed to adequately assess nasolabial cyst recurrence when using techniques other than surgical enucleation.

## CONCLUSION

Nasolabial cysts are infrequent in the general population. Although these cysts may be asymptomatic, the usual presentation is localized swelling, local pain and partial or total nasal obstruction. Computed tomography is the best diagnostic method. Histopathology reveals a non-ciliated columnar epithelium and mucus-producing cells. The treatment of choice is surgical enucleation, which has low recurrence rates.
